# A Defect Detection Method for Functional Membranes in Flexible Sensors for Vibration Monitoring During Glass Substrate Transfer

**DOI:** 10.3390/mi17080933

**Published:** 2026-08-05

**Authors:** Zhuohao Shi, Han Wang, Yibin Chen, Shuai Chen, Daohua Zhan, Weicheng Ou

**Affiliations:** 1 School of Mechanical and Electrical Engineering, Guangdong University of Technology, Guangzhou 510006, China; shizhuohao1@mails.gdut.edu.cn (Z.S.); 2112301497@mail2.gdut.edu.cn (Y.C.); 15913980911@163.com (S.C.); 2 School of Intelligent Manufacturing Equipment, Guangdong Mechanical & Electrical Polytechnic, Guangzhou 510550, China; 1112101024@gdmec.edu.cn; 3 Experimental Teaching Department, Guangdong University of Technology, Guangzhou 510006, China

**Keywords:** functional nanofiber membrane, flexible sensor, defect detection, YOLO, glass substrate transfer, semiconductor display

## Abstract

Vibration monitoring of glass substrate transfer systems is crucial for ensuring the stable operation of Flat Panel Display (FPD) manufacturing equipment. Fabrication defects in the functional nanofiber membrane of flexible vibration sensors can significantly degrade sensing performance and lead to inaccurate monitoring results. To address the challenge of achieving an effective balance between detection accuracy and inference efficiency in such defect-dense scenarios characterized by large variations in defect scale, this paper proposes a novel defect detection model, termed MA-YOLO. The proposed model incorporates four key architectural enhancements: the Multi-level Bidirectional Feature Aggregation Network (MLBAN), the Multi-Receptive Field Adaptive Fusion Module (MRAF), the Morphology-Adaptive Feature Extraction Module (MA-C2f), and the Interactive Dynamic Decoupling Head (IDDH). These components collaboratively improve defect feature extraction, multi-scale feature fusion, and localization performance while maintaining a lightweight architecture and high inference speed. Experimental results on a self-constructed defect dataset demonstrate that MA-YOLO achieves a mean Average Precision (mAP@0.5) of 91.9%, which is a 3.1 percentage point improvement over the baseline model. Moreover, with only 9.15 million parameters and an inference speed of 119.05 FPS, the proposed model exhibits superior overall performance compared with several mainstream and state-of-the-art object detection methods.

## 1. Introduction

**Figure 1:**
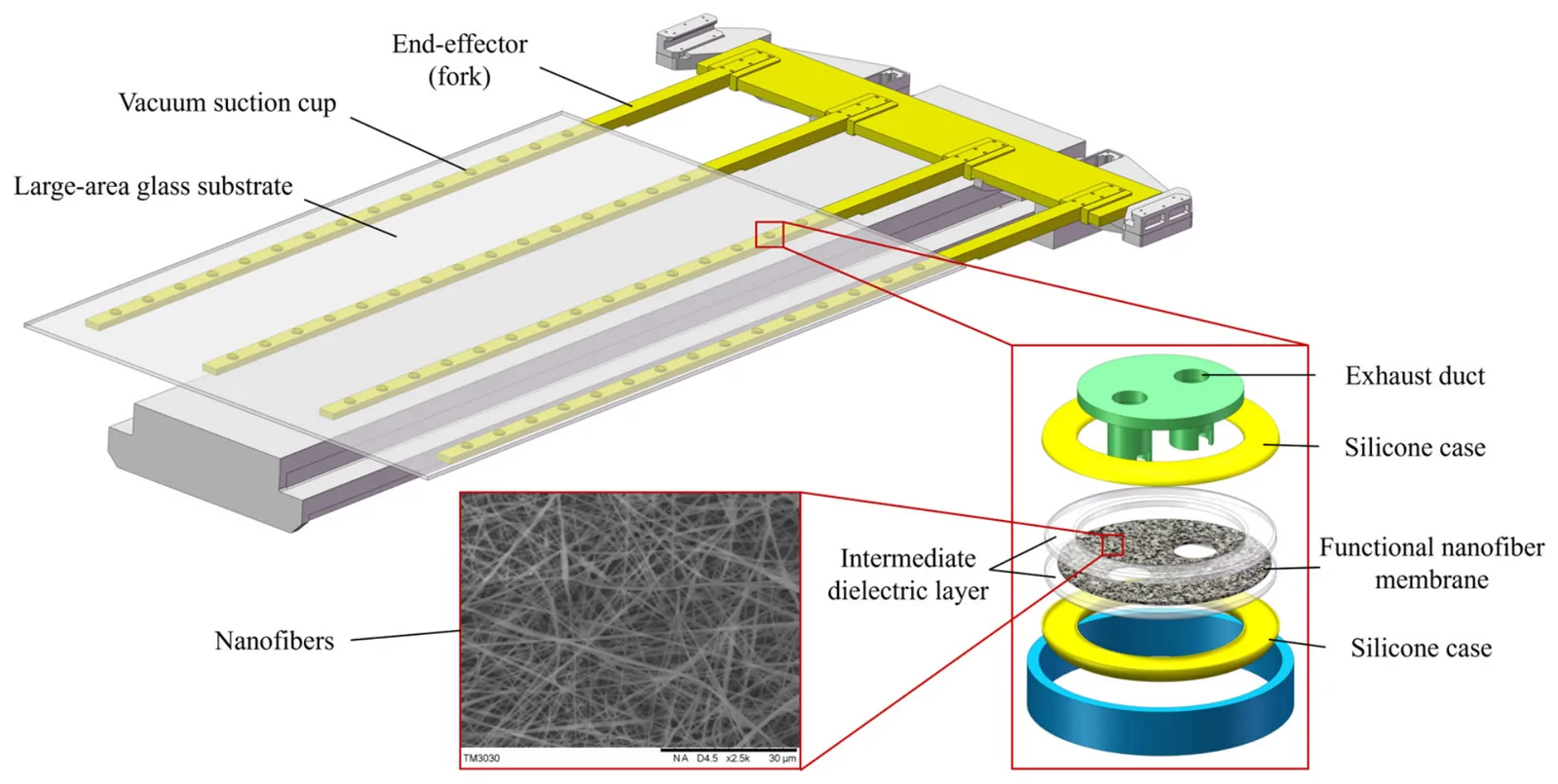
Flexible sensors for vibration monitoring of end-effector (fork).

In advanced Flat Panel Display (FPD) manufacturing equipment, the stability of large-area glass substrate transfer is of critical importance. To prevent substrate slippage, misalignment, or even breakage induced by mechanical vibrations, transfer robots are required to continuously monitor the dynamic vibration responses of substrates during handling operations [1]. Owing to their lightweight structure, high sensitivity, excellent flexibility, and ease of integration, flexible sensors have emerged as an ideal solution for vibration monitoring on robotic end-effectors (forks) [2,3]. Similarly, flexible sensors have also been extended to the field of state recognition in complex mechanical structures, such as through intelligent perception enabled by multimodal large language models [4]. Among the various materials used in flexible sensors, electrospun nanofiber membranes are widely regarded as the preferred choice for constructing the core sensing layer because of their exceptionally high specific surface area, remarkable mechanical flexibility, and outstanding electromechanical response characteristics [5,6]. Figure 1 illustrates the flexible sensor developed by our research group and mounted on the fork for substrate transfer vibration monitoring.

However, the electrospinning process is inherently susceptible to the formation of morphological defects within the nanofiber structure [7]. These defects can significantly deteriorate the mechanical integrity and electrical performance of the functional membrane [8], thereby compromising the stability, reliability, and accuracy of vibration monitoring signals. Consequently, accurate detection of microscopic defects in nanofiber membranes is not only critical for ensuring the sensing performance and long-term reliability of flexible sensors [9], but also serves as a fundamental prerequisite for maintaining the stable operation of FPD manufacturing equipment.

The most prevalent microscopic defects in electrospun nanofiber membranes include bead, splashing, and impurity. These defects typically exhibit substantial variations in size and morphology and often occur in overlapping or densely distributed patterns, making their rapid and accurate identification particularly challenging. Traditionally, defect analysis has relied primarily on manual interpretation of scanning electron microscopy (SEM) images. However, this approach is time-consuming and susceptible to subjective judgment, resulting in limited inspection efficiency and consistency [10]. With the increasing demand for large-scale production and the rising cost of skilled labor, there is a growing need in both industry and academia for automated, efficient, and reliable defect detection methods [11].

Traditional machine learning-based defect detection methods typically rely on manually engineered feature extractors and classifiers. However, such approaches often exhibit limited robustness and poor generalization when confronted with nanofiber defects characterized by diverse morphologies and complex distributions [12,13]. In recent years, convolutional neural networks (CNNs) have demonstrated remarkable success in automated feature learning by extracting hierarchical feature representations directly, thereby effectively overcoming the limitations associated with handcrafted features [14,15,16]. Although Transformer-based object detection frameworks have achieved outstanding performance across a wide range of computer vision tasks [17], their self-attention mechanisms introduce substantial computational complexity and memory consumption [18]. Consequently, they tend to incur considerable computational redundancy when processing high-resolution microscopic images of nanofiber membranes.

Therefore, this study adopts a CNN-based architecture to develop an automated defect detection model for nanofiber membranes. Among existing CNN-based object detection frameworks, the YOLO series has emerged as one of the most widely adopted one-stage detectors owing to its lightweight design and end-to-end regression paradigm. These characteristics make it particularly suitable for real-time industrial inspection applications. However, due to the unique micro-/nano-scale network structure of nanofiber membranes and the highly diverse and complex morphologies of defects, the standard YOLO architecture still faces challenges in accurately detecting multi-scale targets and densely overlapping defects. To overcome these limitations, this study employs YOLOv8 as the baseline framework and systematically optimizes its feature extraction, feature fusion, and classification–localization mechanism to enhance detection performance in complex nanofiber defect scenarios.

The main contributions of this study are summarized as follows:

1. A feature-adaptive extraction and aggregation architecture is proposed for detecting defects with diverse morphologies, scales, and spatial distributions. By integrating Morphology-Adaptive Feature Extraction Module (MA-C2f), Multi-level Bidirectional Feature Aggregation Network (MLBAN), and Multi-Receptive Field Adaptive Fusion Module (MRAF), the proposed architecture enhances feature representation and multi-scale feature fusion.
2. An Interactive Dynamic Decoupling Head (IDDH) is developed to address the loss of shared features in conventional decoupled detection heads. Specifically, a dynamic interaction mechanism is introduced between the classification and localization branches, enabling information exchange while preserving task-specific learning, thereby improving the detection of densely distributed and overlapping nanofiber defects.
3. A microscopic defect dataset for nanofiber membranes is established to support model training and evaluation. Experimental results demonstrate that the proposed MA-YOLO achieves superior accuracy and robustness over the baseline model.

The remainder of this paper is organized as follows. Section 2 presents a review of related work. Section 3 details the construction of the dataset and the architecture of the proposed MA-YOLO network. Section 4 presents and discusses the experimental results. Finally, conclusions are drawn in Section 5.

## 2. Related Works

### 2.1. Microscopic Defect Detection in Electrospun Nanofibers

The electrospinning process is highly susceptible to defect formation owing to the influence of multiple processing parameters [19]. Despite the rapid advancement of deep learning-based visual inspection techniques, automated detection of microscopic defects in nanofiber membranes remains relatively underexplored because of the complexity of defect formation mechanisms and the interdisciplinary nature of the problem [20]. Nevertheless, recent studies have reported encouraging progress in this area.

Carrera et al. [21] trained a model to learn sparse representations of normal nanofiber structures and detected defects by analyzing image patch features. Napoletano et al. [22] combined CNNs with self-similarity analysis to detect anomalies by measuring the similarity between image subregions and normal regions. Although effective for coarse-grained defect detection, the method exhibited reduced accuracy when detecting medium- and fine-grained defects. Kitamura et al. [23] proposed an unsupervised VGG16-based approach that extracts features from both the input image and its reconstructed counterpart, enabling a balanced representation of normal structural compactness and anomalous characteristics. Tao et al. [24] proposed the ViTALnet model, which integrates a Vision Transformer (ViT), global attention, and a pyramid architecture to improve anomaly localization. Lv et al. [25] developed a reconstruction-based method that combines an autoencoder with a generative adversarial network (GAN) and employs U-Net for pixel-wise defect analysis. Bergmann et al. [26] introduced a structural similarity-based perceptual loss function to capture interdependencies among local image regions for anomaly detection. Khare et al. [27] proposed a Mask R-CNN-based framework capable of detecting and quantifying electrospun nanofiber defects at the pixel level.

In summary, although substantial progress has been made in nanofiber defect detection, two major challenges remain. First, the limited scale of available datasets. Owing to the high cost of acquiring scanning electron microscopy (SEM) images, most existing methods rely heavily on the relatively small-scale NanoTWICE dataset, resulting in limited transferability and generalization in practical applications. Second, algorithmic redundancy and computational inefficiency. Existing approaches that combine image reconstruction and difference analysis involve complex processing pipelines, while their high computational cost and slow inference speed hinder deployment in end-to-end defect detection scenarios.

### 2.2. Defect Detection Methods Based on YOLOv8

As a representative single-stage object detection framework, the YOLO series has become one of the most widely adopted deep learning models for industrial defect detection due to its excellent balance between detection accuracy and inference speed. Among its variants, YOLOv8 exhibits outstanding performance in both multi-scale object detection and computational efficiency, making it a strong baseline for industrial inspection tasks [28].

YOLOv8 adopts the canonical “backbone-neck-head” three-stage architecture: the backbone, centered on C2f modules and coupled with the SPPF module, expands multi-scale receptive fields and improves fundamental feature extraction performance; the neck employs a PAN-FAN bidirectional fusion structure to enable multi-scale aggregation of hierarchical features; and the decoupled detection head performs bounding box regression, class prediction, and confidence score estimation separately. Furthermore, this model integrates optimization techniques including the anchor-free detection mechanism, striking a well-balanced trade-off among detection accuracy, inference efficiency, and training stability. Benefiting from its mature modular design, it has become a widely adopted baseline network for industrial defect detection tasks.

To address the challenges encountered in complex industrial environments, numerous studies have introduced targeted improvements to the YOLOv8 architecture. For instance, Gao et al. [29] proposed GBE-YOLOv8 for PCB defect detection. By optimizing the backbone network, incorporating an enhanced feature fusion module, and designing an efficient detection head, the model improves detection efficiency. Nevertheless, this method adopts structured pruning as its core optimization strategy, which compromises its feature extraction capability for complex defects.

To mitigate the effects of complex backgrounds and noise interference, Ma et al. [30] proposed BACG-YOLOv8. By incorporating an attention mechanism into the backbone network and introducing an adaptive multi-scale fusion module, the model demonstrated strong capabilities in defect feature extraction and noise suppression for dark-field microscopy images. However, it exhibits limited efficacy in suppressing false positives induced by structural background interference that highly resembles defect features.

For multi-scale and small-object detection tasks, researchers have primarily focused on network architecture optimization. Wei et al. [31] improved the localization accuracy of tiny defects by introducing a dedicated small-object detection layer, an attention module, and a semantic fusion network. Gui et al. [32] proposed YOLO-ADS for metal surface defect detection, achieving simultaneous improvements in detection accuracy and inference speed on benchmark datasets. While the aforementioned methods bolster multi-scale feature extraction capacity, they fail to concurrently address the inconsistency in feature representation arising from heterogeneous defect morphologies and dense spatial distribution.

In summary, although YOLOv8-based variants have achieved remarkable success across a wide range of defect detection tasks and demonstrated considerable architectural flexibility, most existing modifications target individual detection challenges and lack systematic accommodation for multi-faceted compound problems. When directly applied to nanofiber defect detection tasks, these methods still encounter pronounced bottlenecks. Specifically, the prominent structural background formed by interwoven fibers readily induces false positives; the irregular morphologies and blurred boundaries of defects render conventional convolution features ill-suited for accurate characterization; and defects with cross-scale fluctuations and dense distribution tend to give rise to misalignment between classification and localization features. Accordingly, more targeted multi-dimensional enhancement and optimization mechanisms remain indispensable for this specific application scenario.

## 3. Materials and Methods

This section first presents the collection and construction of the electrospun nanofiber microdefect dataset and introduces the data augmentation strategies adopted to alleviate data scarcity. Subsequently, an improved object detection model, MA-YOLO, is proposed based on the YOLOv8 framework to address the challenges of nanofiber defect detection. Finally, the architecture and key components of the proposed model are described in detail, highlighting their effectiveness in handling irregular, overlapping, and multi-scale defects.

### 3.1. Dataset

**Figure 2:**
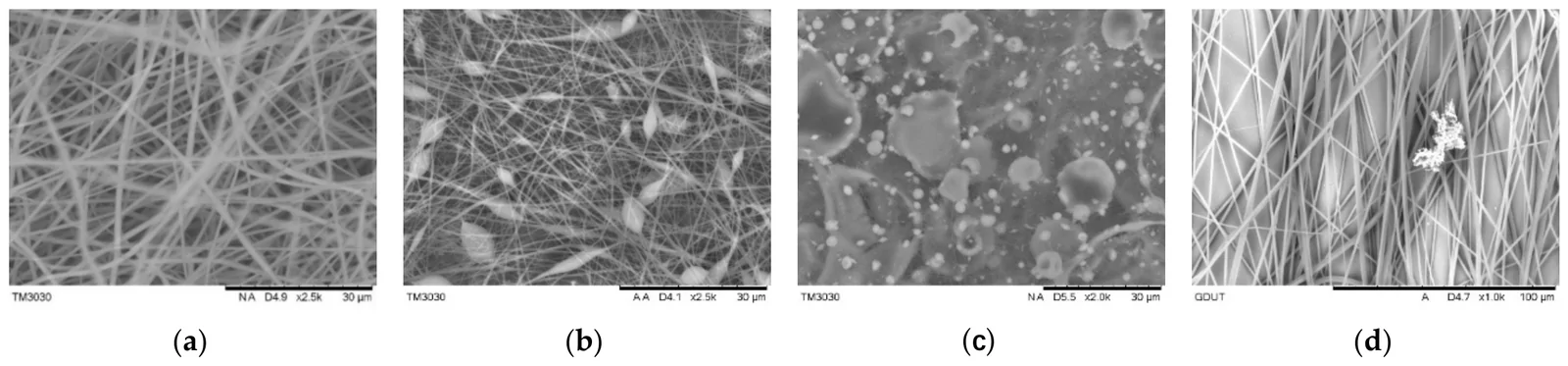
Sample defect types: (**a**) Flawless; (**b**) Bead; (**c**) Splashing; (**d**) Impurity.

SEM images of electrospun nanofiber membranes were acquired in this study. The collected images included both defect-free samples and samples containing three common defect types, namely bead, splashing, and impurity, as illustrated in Figure 2. During dataset construction, only defective samples were retained for subsequent processing and annotation. Since CNN-based defect detection models require a substantial amount of training data, the number of valid defect samples remained limited despite extensive electrospinning experiments. In contrast, a considerable proportion of the acquired images exhibited well-formed nanofiber structures without observable defects and therefore could not be used as positive samples for defect detection training. Consequently, data augmentation was applied to the available defect samples to expand the training set and satisfy the data requirements of model development.

**Figure 3:**
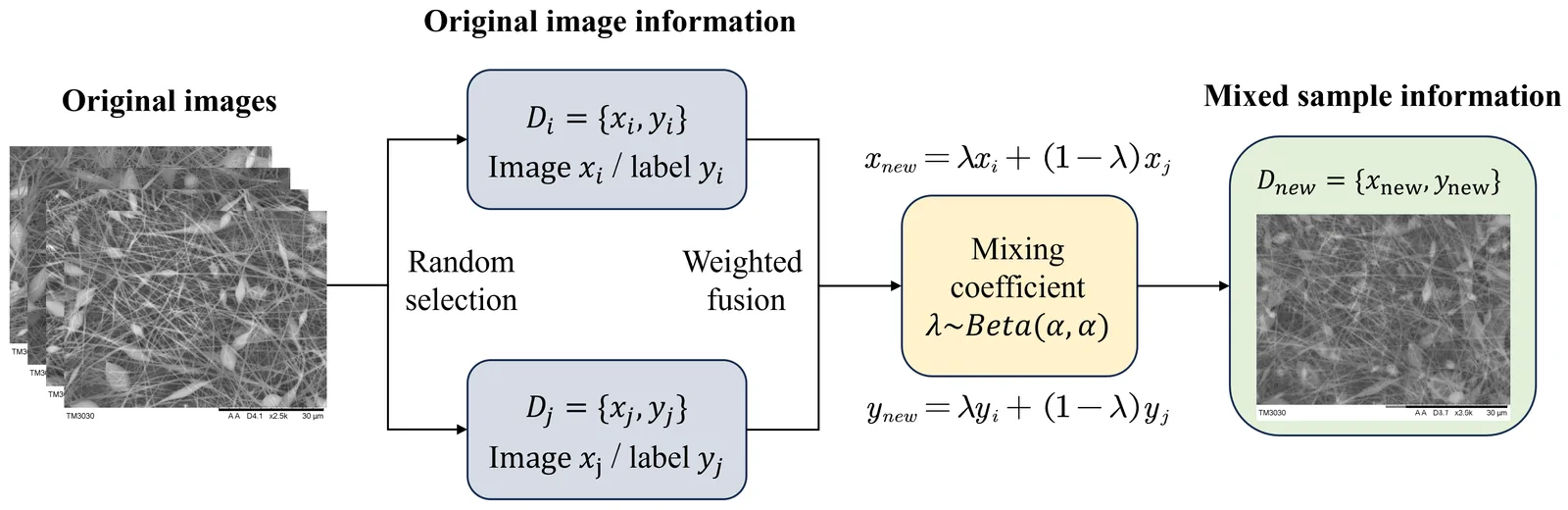
Schematic diagram of the Mixup algorithm.

The nanofiber defects investigated in this study frequently exhibit varying degrees of overlap and occlusion in microscopic images. The Mixup algorithm generates augmented samples by linearly interpolating two randomly selected images and their corresponding labels, as illustrated in Figure 3. The resulting intermediate samples effectively simulate the intertwined and overlapping morphologies commonly observed in real nanofiber defects, thereby enriching the diversity of the training data and providing high-quality samples for model learning. Therefore, Mixup was adopted as the primary data augmentation strategy in this study.

A total of 817 raw SEM images were collected for this study, of which 672 were from defective samples and 145 were from defect-free samples. Among the defective samples, the number of defects classified as “bead,” “splashing,” and “impurity” was 3580, 2912, and 2374, respectively. The originally collected image samples were first randomly partitioned into training, validation, and test sets at a ratio of 8:1:1. Geometric transformations, including rotation and cropping, were then applied to the training set to enhance the model’s robustness to geometric variations. Subsequently, the Mixup algorithm (α = 0.2) was employed to further enrich the training data. Through this two-stage data augmentation strategy, the total number of image samples was increased to 1930.

### 3.2. The Proposed MA-YOLO Model

**Figure 4:**
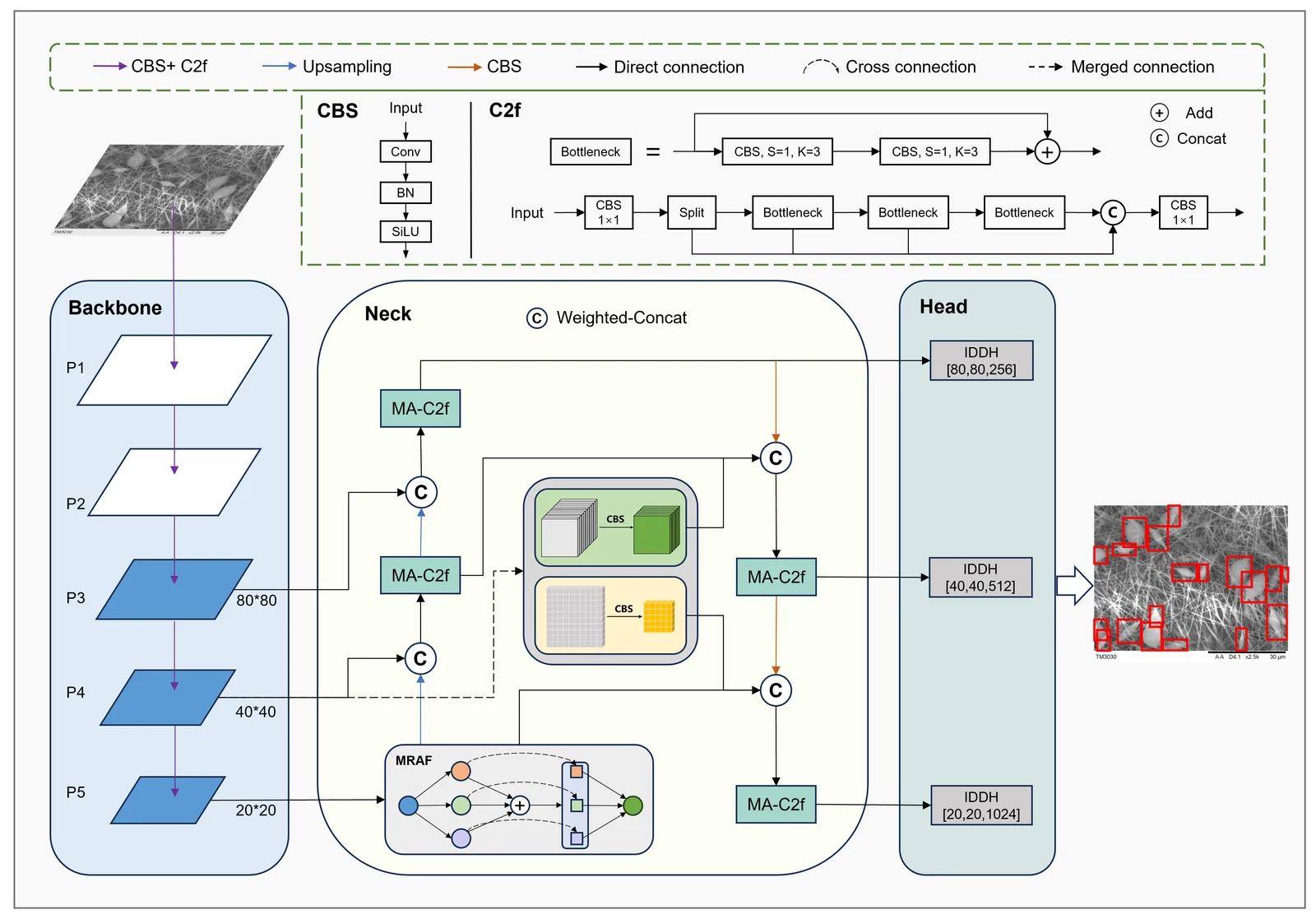
Architecture of the proposed MA-YOLO model.

To address the challenges posed by overlapping, irregularly shaped, and multi-scale nanofiber defects, an improved object detection model, termed MA-YOLO, is proposed based on the YOLOv8 framework. The overall architecture of MA-YOLO is illustrated in Figure 4.

#### 3.2.1. MLBAN

**Figure 5:**
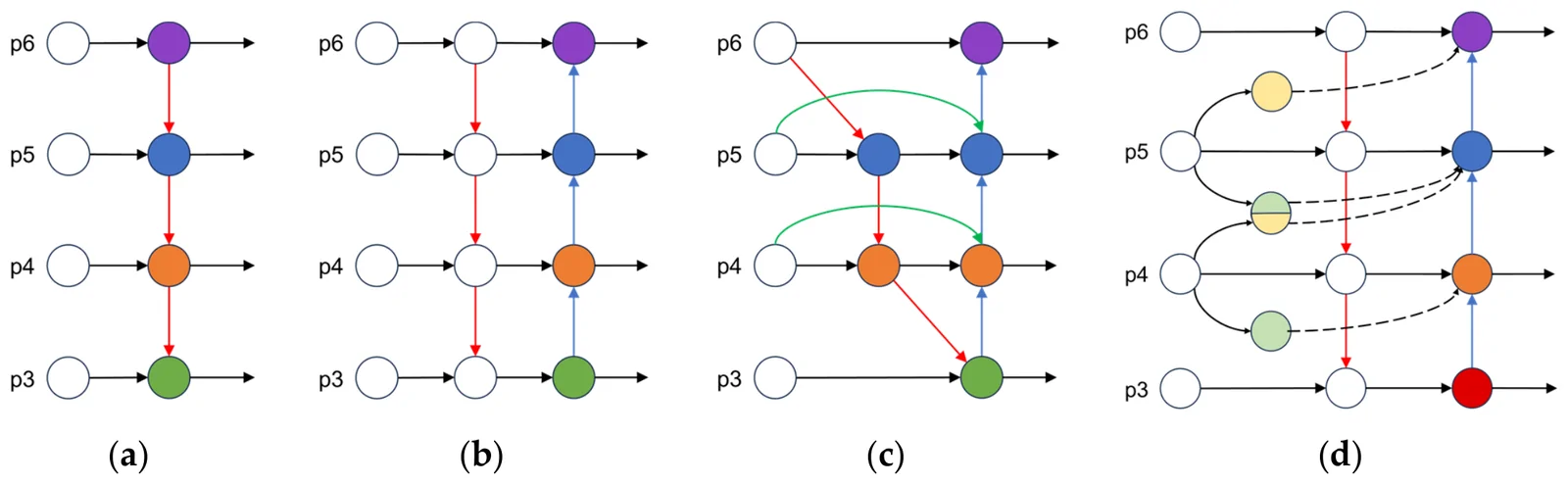
Comparison of feature aggregation network architectures: (**a**) FPN; (**b**) PANet; (**c**) BiFPN; (**d**) MLBAN.

For the defect detection task of nanofiber membranes, the intrinsic size span of three types of defects, beading, spray and impurity can reach tens of times. The imaging characteristics of multiple magnification acquisition of scanning electron microscopes are superimposed, which further expands the fluctuation range of target scales. However, the feature representation capability of a single convolutional layer is inherently limited [33]. Therefore, effective extraction and fusion of multi-level features are essential for improving detection performance. Representative feature aggregation architectures include FPN [34], PANet [35], and BiFPN [36], as shown in Figure 5a–c. FPN adopts a top-down feature fusion strategy, while PANet further enhances feature propagation by introducing a bottom-up path aggregation network. BiFPN extends this design by establishing bidirectional cross-scale connections and employing learnable fusion weights to adaptively integrate features from different paths. However, even if the BiFPN with additional integration path is introduced, the degree of integration of network backbone features is still insufficient. This is mainly because its jump connection is limited to the same-level nodes, and there is still a lack of direct interaction across levels. The details of tiny beads and impurities are continuously diluted in the layer-by-layer sampling, which is easy to cause small targets to be missed. To overcome this limitation, this study proposes a Multi-level Bidirectional Feature Aggregation Network (MLBAN) based on BiFPN, as shown in Figure 5d.

First, MLBAN retains dedicated input nodes to selectively extract information from feature streams, generating feature representations that are more suitable for subsequent fusion. Subsequently, the proposed architecture breaks the constraint of conventional same-level fusion by introducing direct interactions across different feature hierarchies. Specifically, backbone features are spatially downsampled or channel-aligned through transition nodes and then injected via skip connections into adjacent and corresponding layers of the neck network’s bottom-up pathway, thereby enabling cross-level bidirectional feature aggregation. In parallel, same-level feature fusion is performed between the backbone and the neck’s top-down pathway, as well as between the bidirectional pathways within the neck, ensuring comprehensive feature interaction and information preservation. Furthermore, a fast normalized fusion strategy is adopted to adaptively assign weights to different feature paths, thereby enhancing feature fusion efficiency. The corresponding formulation is given as follows:

(1) $$O=\sum_{i}\frac{w_{i}}{\epsilon +\sum_{j}w_{j}}\cdot I_{i}$$

In the equation, $w_{i}$ denotes a trainable weight. To ensure non-negative weight values, a ReLU operation is applied to each $w_{i}$, followed by normalization to constrain the weights within the range of [0, 1]. $I_{i}$ represents the input feature map, where ϵ is a small constant set to 0.0001 to prevent numerical instability during normalization.

#### 3.2.2. MA-C2f

The geometric heterogeneity of three types of defects in nanofiber membranes is prominent: beads are ellipsoidal in different sizes, splashing is dispersed amorphous boundary, and impurities are randomly distributed in the shape of dots and thin strips. Furthermore, defect edges are frequently interwoven and overlapped with the surrounding fiber textures. The standard convolution in the conventional target detection network is limited by the fixed field of sensation and geometry, rendering them incapable of effectively addressing nanofiber defects with heterogeneous morphologies and variable scales. Specifically, such convolutions cannot adaptively conform to defect contours, and substantial fiber background noise is easily incorporated during feature extraction, making it difficult to accurately capture discriminative features of heterogeneous defects. To address this limitation, a Morphology-Adaptive Feature Extraction Module (MA-C2f) is proposed. By incorporating deformable convolutions [37], the module dynamically adjusts sampling locations and modulation weights through learnable offsets and modulation mechanisms. Consequently, the network can better adapt to the geometric boundaries of bead, splashing, and impurity, thereby enhancing defect feature representation while suppressing background interference.

For a standard convolutional operation, the output feature map y can be expressed as:

(2) $$y\left(p_{0}\right)=\sum_{P_{n}\in R}w(p_{n})\cdot x(p_{0}+p_{n})$$

**Figure 6:**
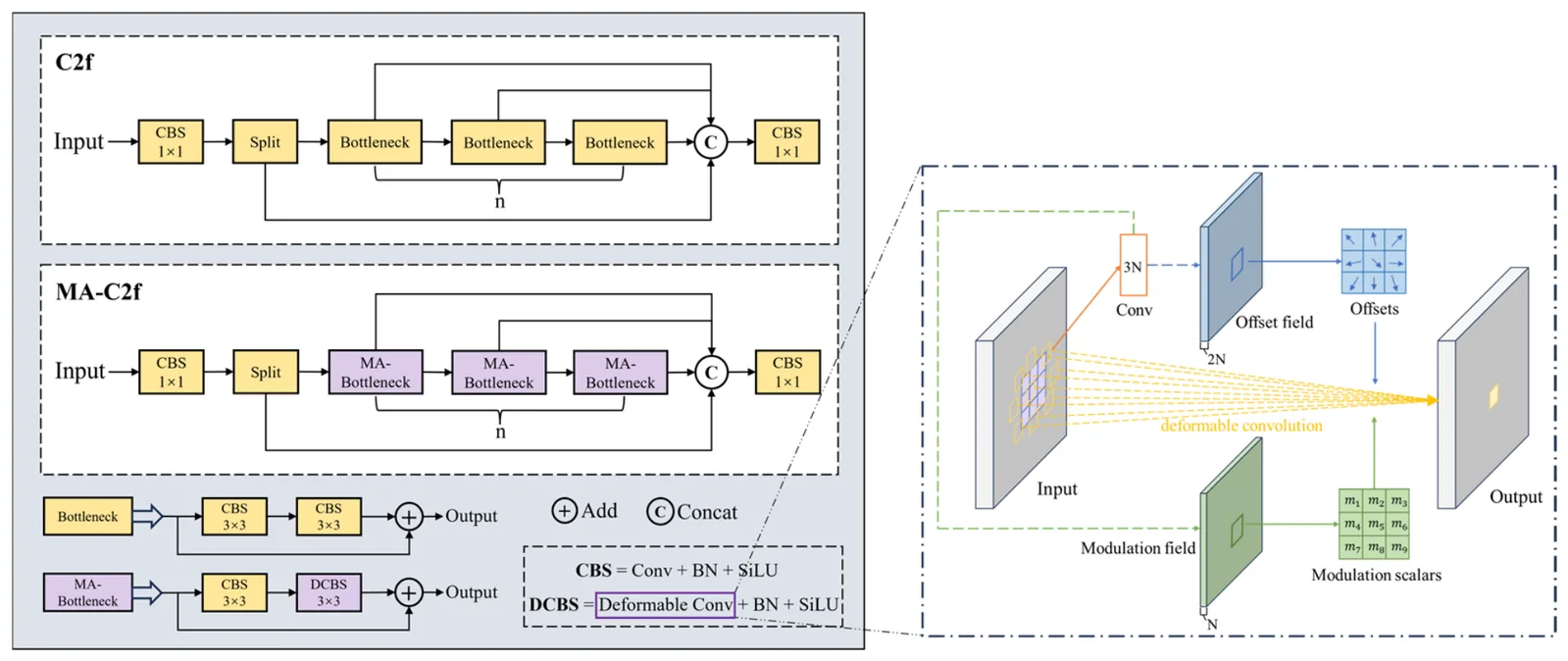
Comparison of the C2f module and the MA-C2f module.

Here, $p_{0}$ denotes a location in the output feature map y, and R defines the sampling grid defined by the convolution kernel size and dilation rate. For example, for a 3 × 3 convolution kernel with a dilation rate of 1, the sampling set can be represented as $R\in \left\{\left(-1,-1\right),\left(-1,0\right)\cdots ,\left(1,0\right),(1,1)\right\}$. $p_{n}$ denotes the n-th sampling location in the sampling grid R, x represents the input feature map, and *w* denotes the convolution weights in R. Deformable convolution extends standard convolution by introducing a learnable offset $∆p_{n}$ and a modulation scalar $∆m_{n}$ for each sample point. The computational principle of deformable convolution is shown on the right side of Figure 6. The output feature map *y* of deformable convolution can be formulated as follows:

(3) $$y\left(p_{0}\right)=\sum_{P_{n}\in R}w(p_{n})\cdot x(p_{0}+p_{n}+∆p_{n})\cdot ∆m_{n}$$

**Figure 7:**
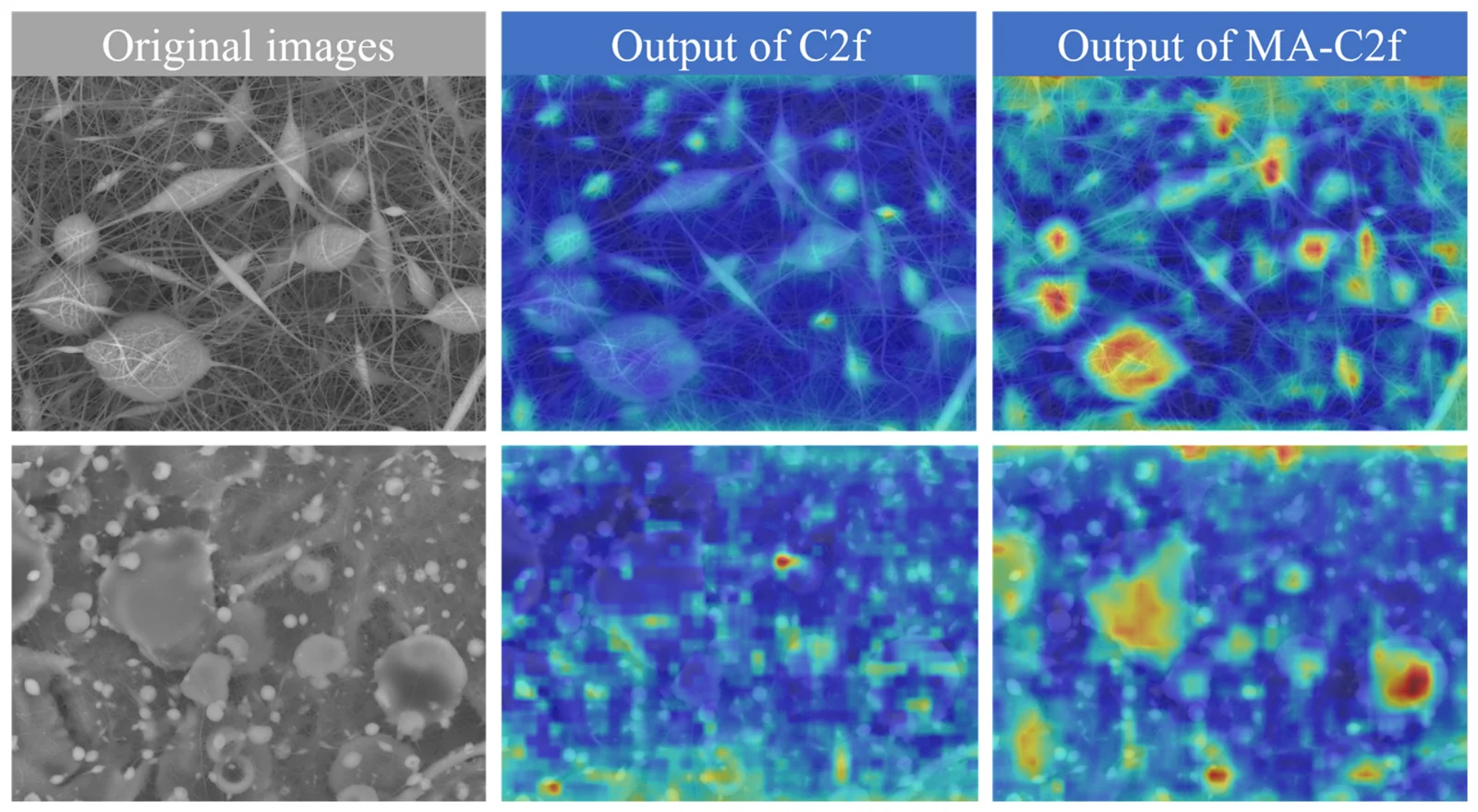
Visual comparison. From left to right: the original input image, the Grad-CAM visualization of C2f, and the Grad-CAM visualization of the proposed MA-C2f. Warmer colors indicate higher activation intensity, whereas cooler colors indicate lower activation intensity.

The structural comparison between the original C2f module and the proposed MA-C2f module is illustrated on the left side of Figure 6. In the proposed MA-C2f, the second 3×3 standard convolution within the Bottleneck block is replaced with a 3×3 deformable convolution. This modification is motivated by the functional characteristics of the two convolutional layers. The first convolution layer primarily performs channel compression, and introducing deformable convolution at this stage may adversely affect the stability of feature representations. In contrast, the second convolution layer is mainly responsible for feature extraction. Replacing it with deformable convolution enables adaptive adjustment of sampling locations, allowing the network to more accurately capture local geometric variations while preserving semantic information in the early stages of feature learning. As illustrated in Figure 7, deformable convolution demonstrates superior capability in capturing and fitting defect boundaries compared with standard convolution.

#### 3.2.3. MRAF

**Figure 8:**
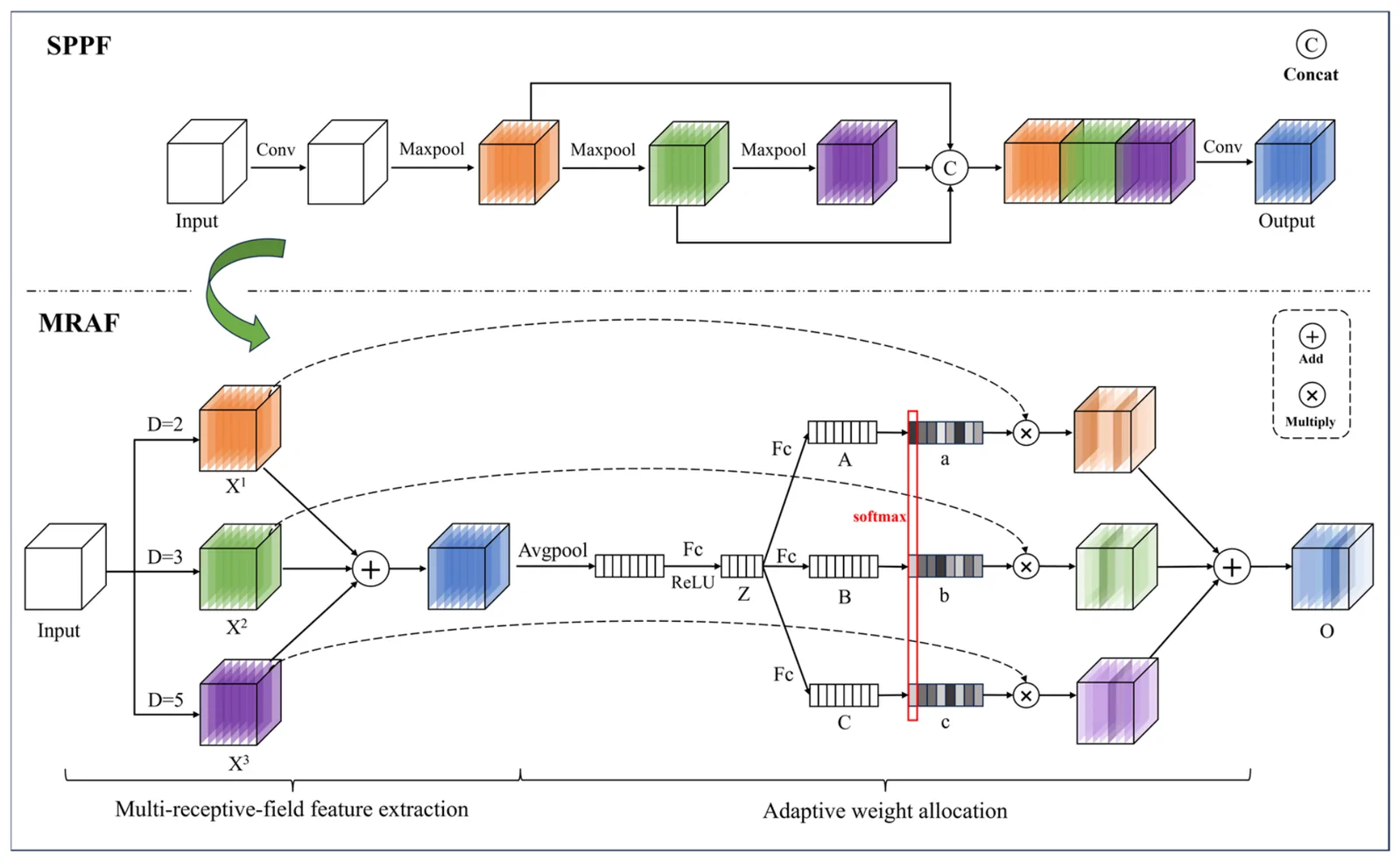
The structure of MRAF module.

Defects in nanofiber membranes span a broad range of scales: impurities and beads exhibit extensive scale variations, while splashing is predominantly present as large-scale diffuse morphologies. Accordingly, features at different scales make unequal contributions to defect detection performance. YOLOv8 employs the SPPF module to perform multi-scale feature fusion, which provides fundamental support for detecting nanofiber defects with significant scale variations. However, its linear fusion mechanism lacks the ability to adaptively model the relative importance of features from different scales, and undifferentiated fusion is easy to cause the key defect features to be diluted by the fiber background. To overcome this limitation, a Multi-Receptive Field Adaptive Fusion (MRAF) module is proposed. The module integrates multi-receptive field feature extraction with adaptive weight allocation, enabling selective enhancement of informative features across different scales. Its architecture is illustrated in Figure 8.

In the multi-receptive field feature extraction stage, MRAF replaces the conventional serial max-pooling strategy with a dilated-group convolution architecture. Although max-pooling enlarges the receptive field, it inevitably sacrifices fine-grained feature details. In contrast, dilated convolution expands the receptive field without reducing spatial resolution, enabling effective capture of multi-scale contextual information. Meanwhile, group convolution significantly reduces model parameters and computational complexity. By employing multiple dilation rates, MRAF can simultaneously extract local texture and global structural features within a single layer, thereby mitigating the loss of subtle defect information.

During the adaptive weight allocation stage, MRAF first aggregates features extracted from the three receptive-field branches through feature fusion and global average pooling to obtain a global feature representation. Next, channel-wise dimensionality reduction is performed using a fully connected layer with a reduction ratio of r = 16, followed by ReLU activation to produce a compact feature vector $Z\in \;R^{d\times 1}$. The compressed feature vector is subsequently mapped through another fully connected layer to generate three scale-specific attention vectors, namely $A\in \;R^{c\times 1}$, $B\in \;R^{c\times 1}$, and $C\in \;R^{c\times 1}$. Inspired by the selective kernel mechanism of SKNet [38], the softmax operator is then applied across the scale dimension to normalize the attention weights, ensuring that the weights assigned to features of different receptive fields within the same channel sum to one. The corresponding formulation is given as follows:

(4) $$a_{c}=\frac{e^{A_{c}}}{e^{A_{c}}+e^{B_{c}}+e^{C_{c}}},b_{c}=\frac{e^{B_{c}}}{e^{A_{c}}+e^{B_{c}}+e^{C_{c}}},\;c_{c}=\frac{e^{C_{c}}}{e^{A_{c}}+e^{B_{c}}+e^{C_{c}}}$$

In the equation, $a_{c}$ is the c-th element of the weight vector a, representing the weight proportion of the multiscale features of feature A in the c-th channel; $b_{c}$ and $c_{c}$ are defined similarly. $A_{c}$ represents the feature in the c-th channel of the feature vector; $B_{c}$ and $C_{c}$ are defined similarly.

Finally, the original features at the three scales are multiplied by their assigned weights and then summed to obtain the final feature map O:

(5) $$O_{c}=a_{c}\cdot X_{c}^{1}+b_{c}\cdot X_{c}^{2}+c_{c}\cdot X_{c}^{3},\;a_{c}+b_{c}+c_{c}=1$$

Here, $O\;=\;[O_{1},\;O_{2},O_{3}]$; $X_{c}^{1}$ represents the feature from the c-th channel of the original feature $X^{1}$, and $X^{2}$ and $X^{3}$ are defined analogously.

#### 3.2.4. IDDH

**Figure 9:**
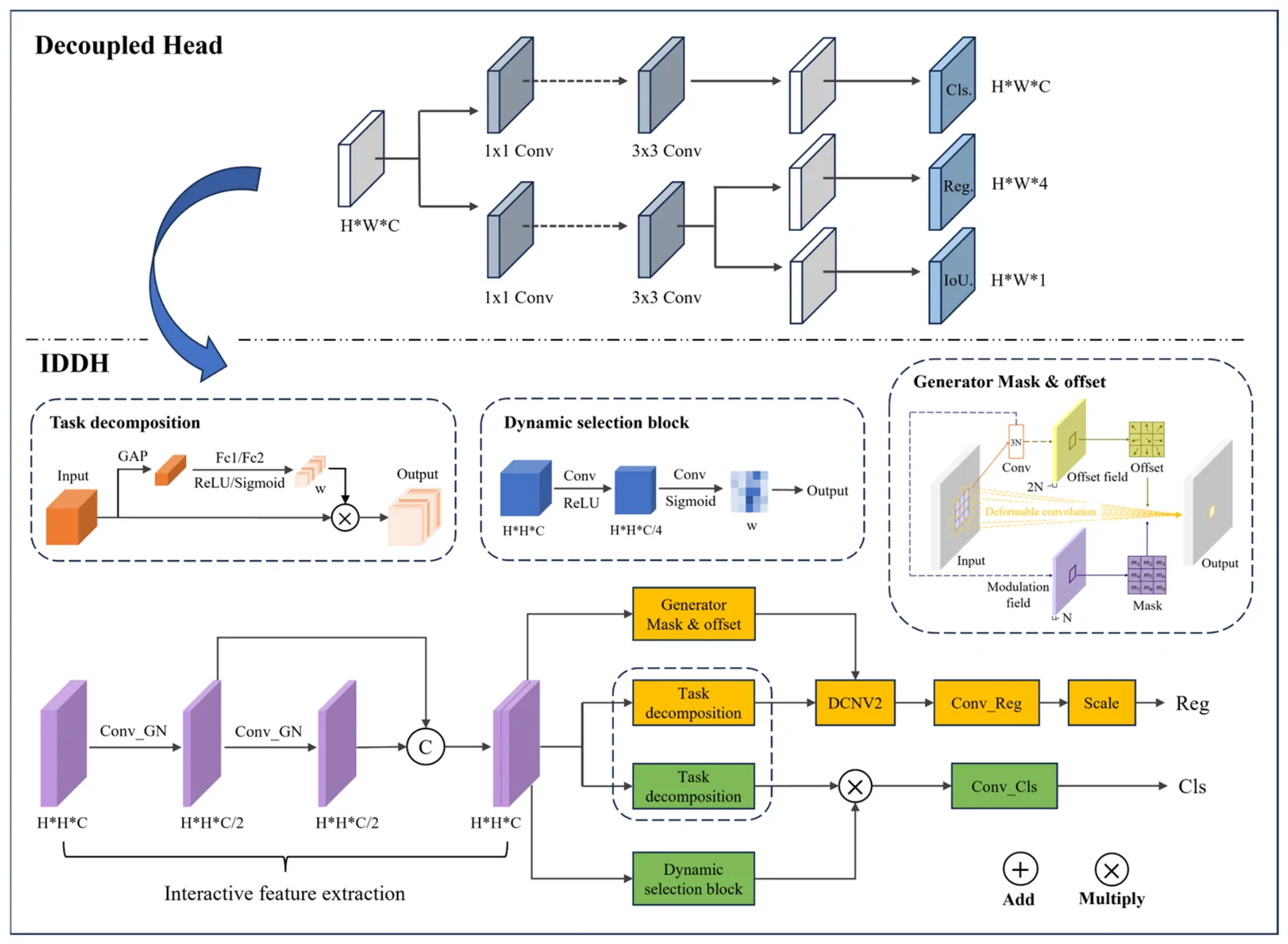
The structure of IDDH.

The defects on the surface of nanofiber membranes are often distributed in dense clusters, and the defects have the characteristics of fuzzy boundary and irregular shape. The coupling degree between the classification features and positioning features of defects is high, and the requirements for the dual branch cooperation ability are much higher than the general target detection scenario. YOLOv8 employs a decoupled detection head to separate classification and localization into independent branches, mitigating the task conflicts present in conventional coupled heads. Nevertheless, the design has obvious adaptation short board in this task, and the independent operation of the two branches lacks two-way feature interoperability, which not only increases the amount of model parameters, but also cannot adapt to the dense distribution and fuzzy boundary characteristics of fiber defects. The problem of inconsistent feature expression is easy to occur, which can cause the missing detection of dense micro defects and the false detection of similar texture defects, which seriously limits the detection accuracy. To overcome these limitations, an Interactive Dynamic Decoupled Head (IDDH) is proposed. By introducing a feature interaction bridge between the classification and localization branches, IDDH strengthens inter-task collaboration and enhances the detection performance of complex nanofiber defects. Its overall architecture is shown in Figure 9.

IDDH first employs a shared two-layer Conv_GN (Convolution + GroupNorm) module to extract task-interactive features. Specifically, the shallow convolution layer captures fine-grained local details, whereas the deeper convolution layer encodes higher-level semantic information. Through hierarchical feature aggregation, the effective receptive field is progressively expanded. This shared design promotes information exchange between the classification and localization branches via parameter sharing while enabling task-specific feature representations through multi-level feature fusion. Furthermore, GroupNorm regularizes feature distributions by channel grouping, thereby enhancing the classification and localization capabilities of the detection head [39].

After obtaining the interactive features, conflicts still exist between the classification and localization tasks due to their distinct feature preferences. To address this issue, a task decomposition module is introduced to separate the shared features and generate task-specific feature representations for the two tasks. The module performs feature decomposition through dynamic weight adjustment. Specifically, global average pooling is first applied to the input features to capture global contextual information, followed by a fully connected layer and a Sigmoid activation function to generate task-aware channel attention weights. The corresponding formulation is given as follows:

(6) $$w_{channel}=\sigma \left(F_{c2}\left(\delta \left(F_{c1}\left(x_{in}\right)\right)\right)\right)$$

(7) $$X_{out}^{k}=w_{channel}^{k}\cdot X_{in}^{k},\;\forall k\in \left\{1,2,\cdots ,N\right\}$$

where $w_{channel}$ denotes the channel attention weights; $F_{c1}$ and $F_{c2}$ are two fully connected layers; δ and σ denote the ReLU and Sigmoid activation functions, respectively; and $x_{in}$ is obtained by applying global pooling to the interactive feature map $X_{in}$. $w_{channel}^{k}$ denotes the k-th element of the channel attention weights, while $X_{in}^{k}$ and $X_{out}^{k}$ represent the k-th channel features of the interaction feature map and the task-specific feature map, respectively.

Although the task decomposition module adaptively allocates features at the channel level, it does not explicitly model the distinct spatial requirements of classification and localization tasks. To address this issue, IDDH further introduces a dual-branch spatial calibration mechanism. The localization branch employs deformable convolution to dynamically learn geometric offsets and spatial modulation masks, enabling adaptive focus on key regions of the target bounding box. In parallel, the classification branch extracts category-discriminative features through two convolutional layers. The resulting spatial attention weights are generated via a Sigmoid function and applied to the original features through element-wise multiplication, thereby enhancing responses in target regions while suppressing low-confidence background interference. The corresponding formulation is given as follows:

(8) $$w_{spatial}=\sigma \left({Conv}_{2}\left(\delta \left({Conv}_{1}\left(X_{in}\right)\right)\right)\right)$$

(9) $$X_{class}^{k}=w_{spatial}\cdot X_{out}^{k},\;\forall k\in \left\{1,2,\cdots ,N\right\}$$

where $w_{spatial}$ represents the spatial attention weights, $X_{in}$ represents the task interaction feature map, and ${Conv}_{1}$ and ${Conv}_{2}$ denote 1 × 1 and 3 × 3 convolution layers, respectively. $X_{class}^{k}$ represents the k-th channel of the classification feature map after spatial calibration; $X_{out}^{k}$ denotes the k-th channel of the task-specific classification features after task decomposition.

Finally, the localization and classification features are forwarded to their respective prediction layers to produce bounding-box regression outputs and classification scores. In addition, a Scale layer is introduced to adaptively rescale the regression features, thereby accommodating the target-scale variations encountered by different detection heads.

## 4. Experiments and Discussions

To validate the effectiveness of the proposed method, the MA-YOLO model was trained and evaluated on the Nanofiber Defects dataset. All experiments were conducted using PyTorch 1.13.0 with Python 3.8.18 on a workstation equipped with an Intel Core i9-13900KF CPU, an NVIDIA GeForce RTX 4090 GPU, and 64 GB of RAM. During training, stochastic gradient descent (SGD) was adopted as the optimizer with an initial learning rate of 0.01, a batch size of 32, an input image size of 640 × 640, a momentum coefficient of 0.937, a weight decay coefficient of 0.0005, and 300 training epochs.

### 4.1. Evaluation Metrics

To comprehensively evaluate the detection performance of the proposed model, mean Average Precision (mAP), Recall, floating-point operations (FLOPs), Params, and frames per second (FPS) were adopted as evaluation metrics. IoU was employed to measure the overlap between predicted bounding boxes and ground-truth annotations. A prediction was considered correct when its IoU exceeded a predefined threshold; otherwise, it was regarded as an incorrect detection. Based on the IoU criterion, Precision and Recall were calculated as follows:

(10) $$Precision=\frac{TP}{TP+FP}$$

(11) $$Recall=\frac{TP}{TP+FN}$$

where TP, FP, and FN denote the numbers of true positives, false positives, and false negatives, respectively. Precision evaluates the reliability of positive predictions, whereas Recall measures the model’s ability to detect actual defect instances. mAP is computed as the mean of the Average Precision (AP) values over all defect categories. For each category, AP corresponds to the area under the Precision–Recall (PR) curve. A higher mAP value indicates better overall detection performance. The formulations of AP and mAP are expressed as follows, where p(r) denotes the Precision value at Recall, and N is the total number of defect categories.

(12) $$AP=\int_{0}^{1}p\left(r\right)dr$$

(13) $$mAP=\frac{1}{N}\sum_{i=1}^{N}AP_{i}$$

In summary, mAP@0.5 and mAP@0.5:0.95 are used to evaluate detection accuracy; FLOPs and Params are adopted to assess computational complexity and model size, respectively. FPS assesses inference speed, with a timing scope covering the full pipeline of image preprocessing, model forward inference, and post-processing, and the batch size is set to 16 for all speed tests.

### 4.2. Ablation Experiment

#### 4.2.1. Ablation Study on Dilation Rates in MRAF

The MRAF module employs parallel multi-branch dilated convolutions to extract multi-scale features, where the dilation rate configuration directly influences receptive field expansion and feature coverage continuity. To identify the optimal configuration, six representative dilation rate schemes were designed according to the principles of hybrid dilated convolution [40]. The evaluated schemes were [1, 2, 4], [2, 3, 5], [1, 3, 7], [3, 6, 9], [1, 4, 7], and [1, 5, 9]. Among them, the latter two configurations were specifically designed to better accommodate the cross-scale characteristics of the three nanofiber defect categories.

The experimental results are shown in Table 1. The differences in the number of parameters and computational complexity among the various schemes were all below 1%, while the inference speed remained stable within the range of 120–130 FPS. Given the negligible impact of different configurations on model complexity, these metrics are omitted from the table. In terms of detection accuracy, the dilation-rate combination [2, 3, 5] achieved the best performance, attaining an mAP@0.5 of 90.2% and an mAP@0.5:0.95 of 69.5%. Specifically, the mAP@0.5 for impurity defects reached 89.7%, representing an improvement of 1.1 percentage points over the second-best configuration. The mAP@0.5 values for bead and splashing defects were 84.3% and 96.8%, respectively, both exceeding the average performance across all evaluated configurations. These results indicate that the [2, 3, 5] dilation rate scheme effectively accommodates defect features at different scales and exhibits superior adaptability to multiscale nanofiber defect detection. These findings demonstrate the superior adaptability of the [2, 3, 5] dilation rate configuration to multi-scale nanofiber defects.

**Figure 10:**
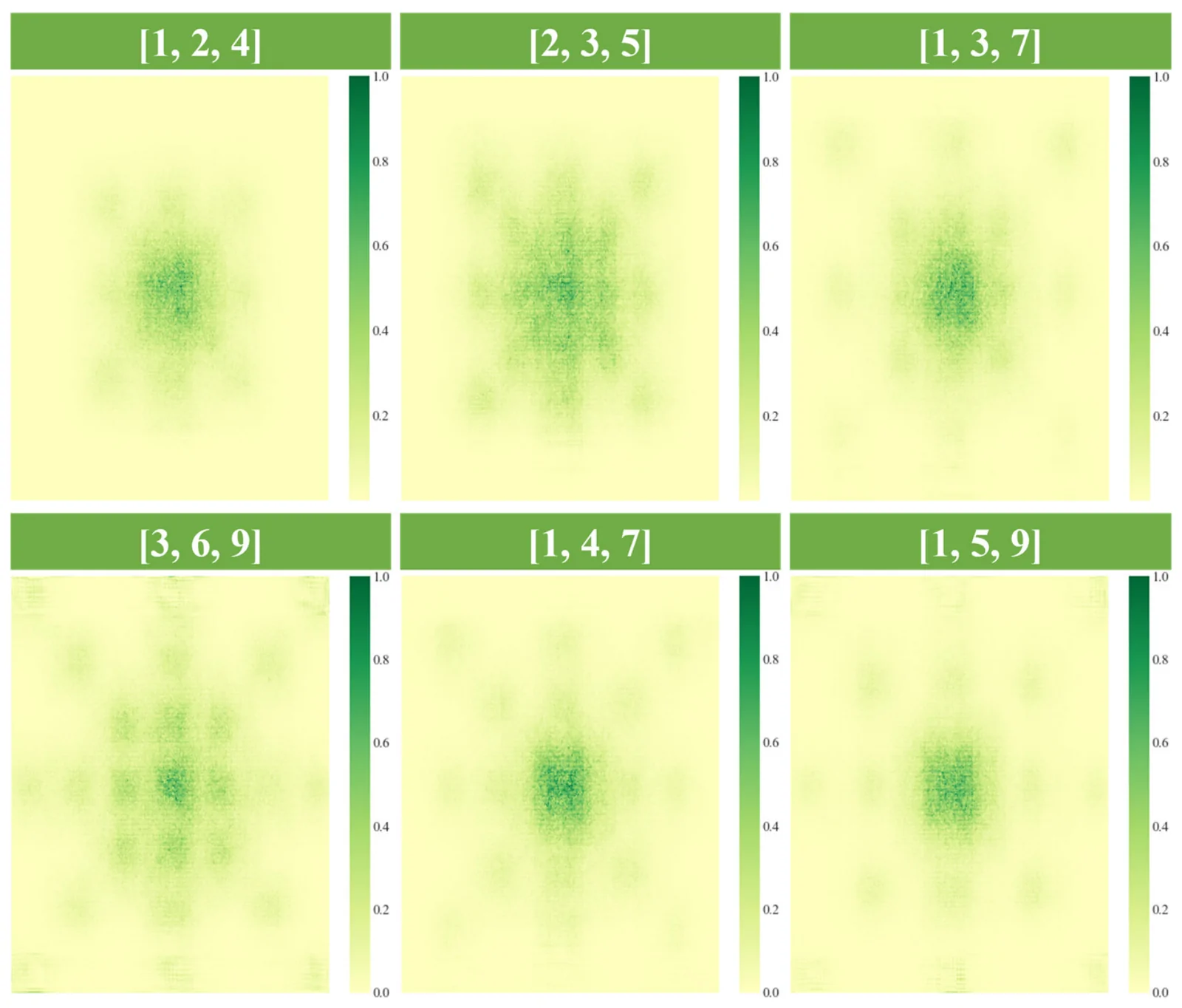
ERF distributions for different dilation rate combinations.

To further validate the superiority of the dilation rate configuration [2, 3, 5], the Effective Receptive Field (ERF) visualization method proposed by Ding et al. [41] was employed to analyze the ERF distributions of the feature layer within the MRAF module under different dilation rate settings. The results are presented in Figure 10, where darker regions indicate larger effective receptive fields. A comparison of the six configurations reveals that the [1, 2, 4] scheme produces the smallest effective receptive field, resulting in insufficient spatial coverage and limited feature extraction capability. Although configurations such as [1, 3, 7] and [3, 6, 9] provide broader receptive field coverage, the excessively large dilation rate intervals lead to discontinuous receptive field distributions, thereby weakening the spatial correlations among defect features. In contrast, the [2, 3, 5] configuration achieves a more favorable balance between receptive field coverage and continuity while maintaining comparable computational cost. As a result, it strengthens multiscale feature correlations and effectively reduces the risks of both false-positive and false-negative detections.

#### 4.2.2. Comparison Between MLBAN and Mainstream Feature Fusion Architectures

To validate the effectiveness of the proposed MLBAN, comparative experiments were conducted using PANet and BiFPN as representative feature fusion architectures. The results are shown in Table 2. PANet, as the original structure of the baseline model, achieved an mAP@0.5 of 88.8%, an mAP@0.5:0.95 of 68% and an inference speed of 128.21 FPS. Although BiFPN maintains a comparable number of parameters and floating-point operations to the baseline, it yields a slightly higher inference speed, while its overall detection accuracy decreases to 88.3% mAP@0.5 and 67.7% mAP@0.5:0.95. In particular, a noticeable performance degradation is observed in the detection of small-scale defects, such as impurities.

In contrast, MLBAN achieves an mAP@0.5 of 89.9%, an mAP@0.5:0.95 of 69.1%, both representing a 1.1 percentage-point improvement over the baseline PANet, while introducing only a marginal increase in model complexity in terms of parameters and computational cost. In particular, the improvement in impurity defect detection is the most significant, reaching 2.9 percentage points, while the performance on splashing defects is also improved. Moreover, MLBAN maintains a higher inference speed than PANet, achieving simultaneous improvements in both detection accuracy and inference efficiency. Overall, these results indicate that MLBAN is more suitable for handling the multi-scale and densely distributed characteristics of nanofiber defects.

#### 4.2.3. Ablation Study on the Position of MA-C2f

**Figure 11:**
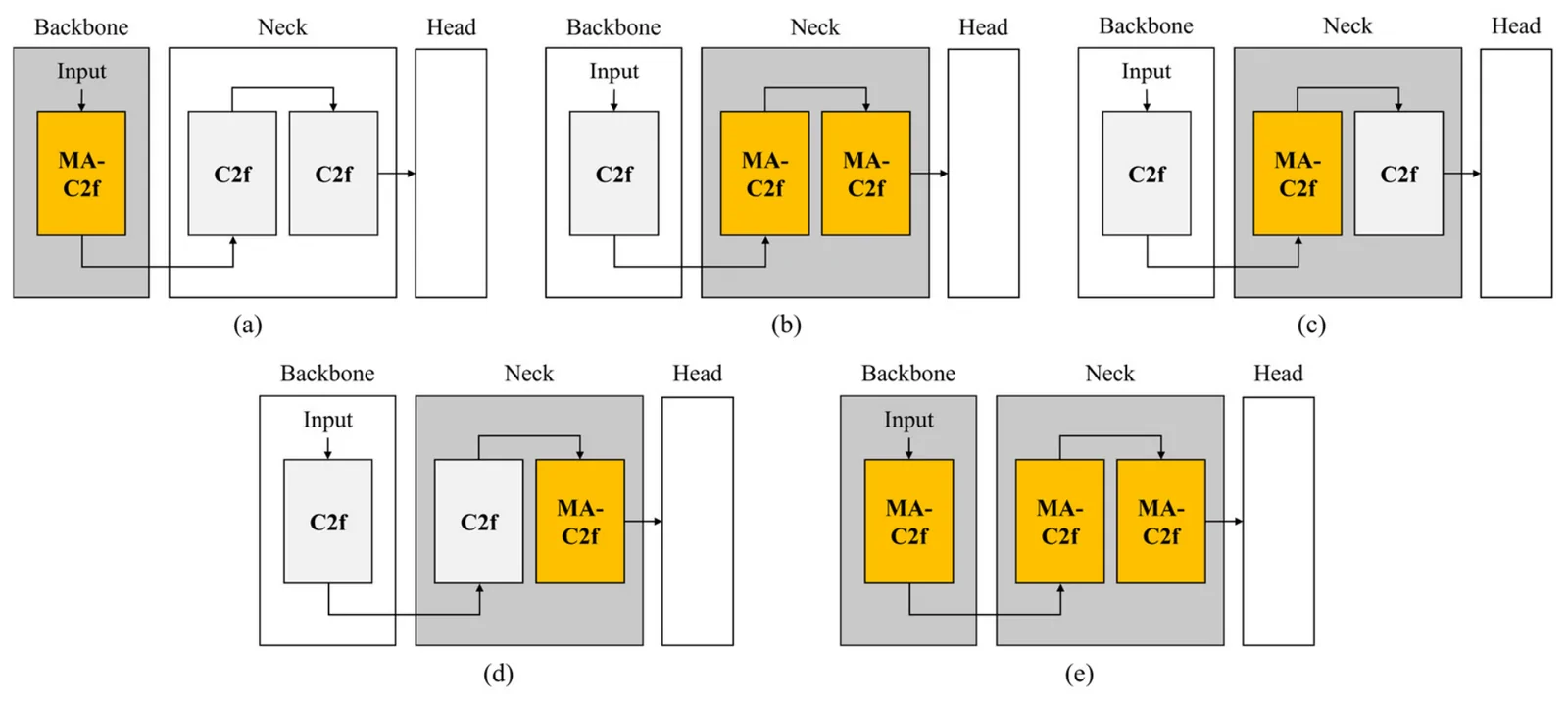
Schematic illustration of MA-C2f deployment across different network layers: (**a**) the backbone; (**b**) the neck; (**c**) the upsampling path of the neck; (**d**) the downsampling path of the neck; (**e**) the full-network integration.

To investigate the performance variations in the MA-C2f module across different network layers, ablation experiments are conducted to analyze the impact of its placement on irregular feature representation. As illustrated in Figure 11, five deployment strategies are evaluated.

As shown in Table 3, the performance of MA-C2f varies significantly with its deployment locations within the network.

Method a: Although this configuration achieves an mAP@0.5 of 89.4% and an mAP@0.5:0.95 of 69.5%, the adaptive feature modeling capability of deformable convolution cannot be fully exploited due to the constraints of high-resolution backbone features and the fixed hierarchical structure of the network. Moreover, the increased computational overhead leads to a reduced inference speed of 111.11 FPS.

Method b: This configuration achieves the highest overall performance, with an mAP@0.5 of 89.8%, an mAP@0.5:0.95 of 70.0% and an inference speed of 119.05 FPS, demonstrating an optimal balance between detection accuracy and computational efficiency. Positioned at a key stage of multi-scale feature fusion, this placement allows deformable convolutions to fully exploit their ability to model local geometric variations. In particular, the mAP@0.5 for splashing and impurity defects reaches 97.3% and 87.5%, respectively, both surpassing the results of other deployment strategies.

Methods (c) and (d) yield an mAP@0.5 of 88.4%, below the baseline, indicating limited effectiveness at these positions. Method (e) achieves an mAP@0.5 of 88.8% and an mAP@0.5:0.95 of 69.1%, while its inference speed drops to 104.17 FPS with elevated computational overhead. Such results indicate that densely deployed deformable convolutions tend to introduce feature space noise, which in turn impairs model robustness. Overall, the MA-C2f module performs best when placed in the neck network.

#### 4.2.4. The Ablation Experiment of Optimization Strategies

To evaluate the effectiveness of each proposed module, ablation experiments were conducted by progressively incorporating the MLBAN, MRAF, IDDH, and MA-C2f modules into the baseline model. The results are reported in Table 4.

The ablation experiments adopt YOLOv8 as the baseline model, which achieves an mAP@0.5 of 88.8%, with 11.13M parameters and an inference speed of 128.21 FPS.

After integrating the MLBAN module, the mAP@0.5 increases to 89.9% (+1.1%), indicating that multi-level feature aggregation effectively enhances the discriminative power of defect representations. Interestingly, the inference speed improves rather than decreases, as adaptive weight-guided feature selection reduces redundant computations in later network stages, enabling simultaneous improvements in accuracy and efficiency.

Furthermore, replacing the original SPPF module with MRAF increases mAP@0.5 to 90.4%, while simultaneously reducing both the number of parameters and FLOPs. This demonstrates that the proposed multi-branch dilated convolution and grouped convolution design not only improves computational efficiency but also strengthens multi-scale feature fusion for defect representation.

After replacing the standard C2f with MA-C2f in the neck region, the mAP@0.5 increases to 91.0% (+0.6%), demonstrating that deformable convolutions better adapt to irregular nanofiber defect features through dynamic sampling.

Finally, replacing the original decoupled head with IDDH improves mAP@0.5 to 91.9% (+0.9%) while reducing the number of parameters to 9.15M. This demonstrates that the proposed IDDH effectively reduces parameter redundancy and enhances the interaction between classification and regression branches.

Overall, the final model integrating all proposed modules yields a 3.1 percentage point improvement in mAP@0.5 and a 4.0 percentage point improvement in mAP@0.5:0.95 relative to the baseline, while reducing the number of parameters by 17.8%. These results demonstrate that the proposed improvements significantly enhance defect detection performance in dense nanofiber materials while maintaining a favorable balance between model compactness and rapid inference performance, confirming the effectiveness and synergistic benefits of the overall framework.

**Figure 12:**
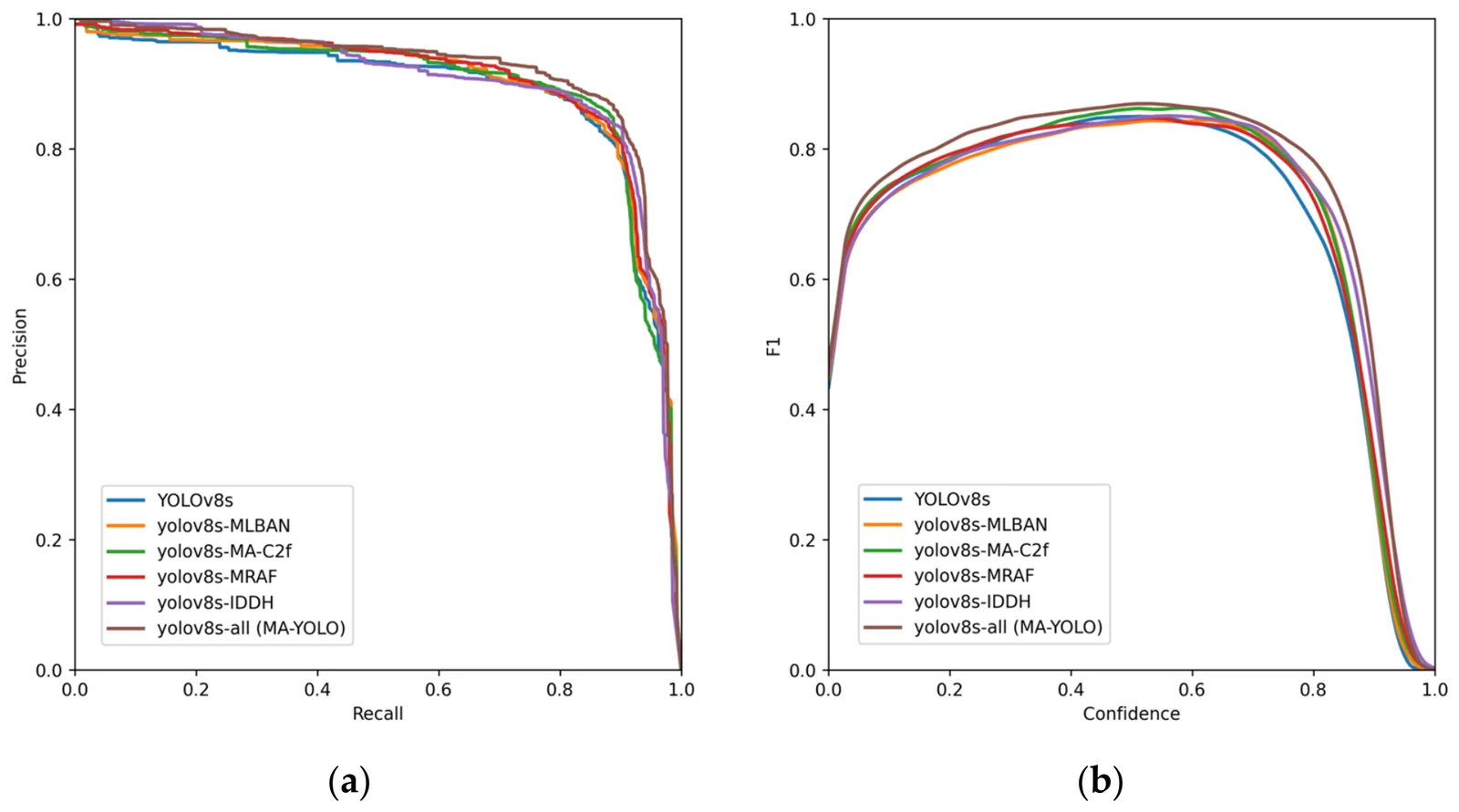
Comparison of detection performance between the proposed model and baseline model: (**a**) P-R curve; (**b**) F1-Confidence curve.

The improved model demonstrates a superior balance between precision and recall, as illustrated in Figure 12. The precision–recall (P-R) curve shows that the area under the curve of the proposed model is significantly higher than that of the baseline, indicating that the synergistic effects of multiple optimization strategies enhance feature representation and classification performance, thereby improving overall detection accuracy. Furthermore, the F1–confidence curve reveals that the proposed model consistently outperforms the baseline across all confidence thresholds, achieving a higher peak F1 score of 0.87 at 0.58, compared with 0.85 at 0.469 for the baseline model. These results confirm the superior overall performance of the proposed approach for defect detection.

### 4.3. False Positive Test of Normal Nanofiber Membrane

Existing object detection evaluation frameworks primarily rely on defect samples to calculate performance metrics such as precision and recall, where prediction results are matched and verified against manually annotated ground-truth bounding boxes. However, for practical nanofiber defect detection systems, achieving high defect detection accuracy alone is insufficient. Such systems must also possess the capability to distinguish between normal fiber textures and actual defects. Therefore, the false-positive level on defect-free membrane samples has become one of the key indicators for evaluating the practical applicability and reliability of the detection system.

To address this issue, an independent defect-free image testing subset was constructed in this section. All samples were completely independent of the original dataset and were not involved in model training or hyperparameter optimization. The subset consisted of 48 original SEM images containing no defects, covering normal randomly distributed nanofiber morphologies obtained under different spinning process parameters and magnification conditions. This subset was used as a pure negative test set. Three evaluation metrics, including the total number of predicted defect bounding boxes, the average number of false positives per image (FPI), and the image-level false-positive rate (FPR), were employed to quantitatively evaluate the false detection performance of the model on defect-free membranes. The calculation methods are given as follows:

(14) $$FPI=\frac{N_{fp}}{N_{img}}$$

(15) $$FPR=\frac{N_{fp-img}}{N_{img}}$$

where $N_{img}$ is the total number of flawless film images; $N_{fp}$ is the total number of effective prediction frames output by the model in all flawless film images; $N_{fp-img}$ is the number of flawless film images that generate at least one false positive prediction box.

**Figure 13:**
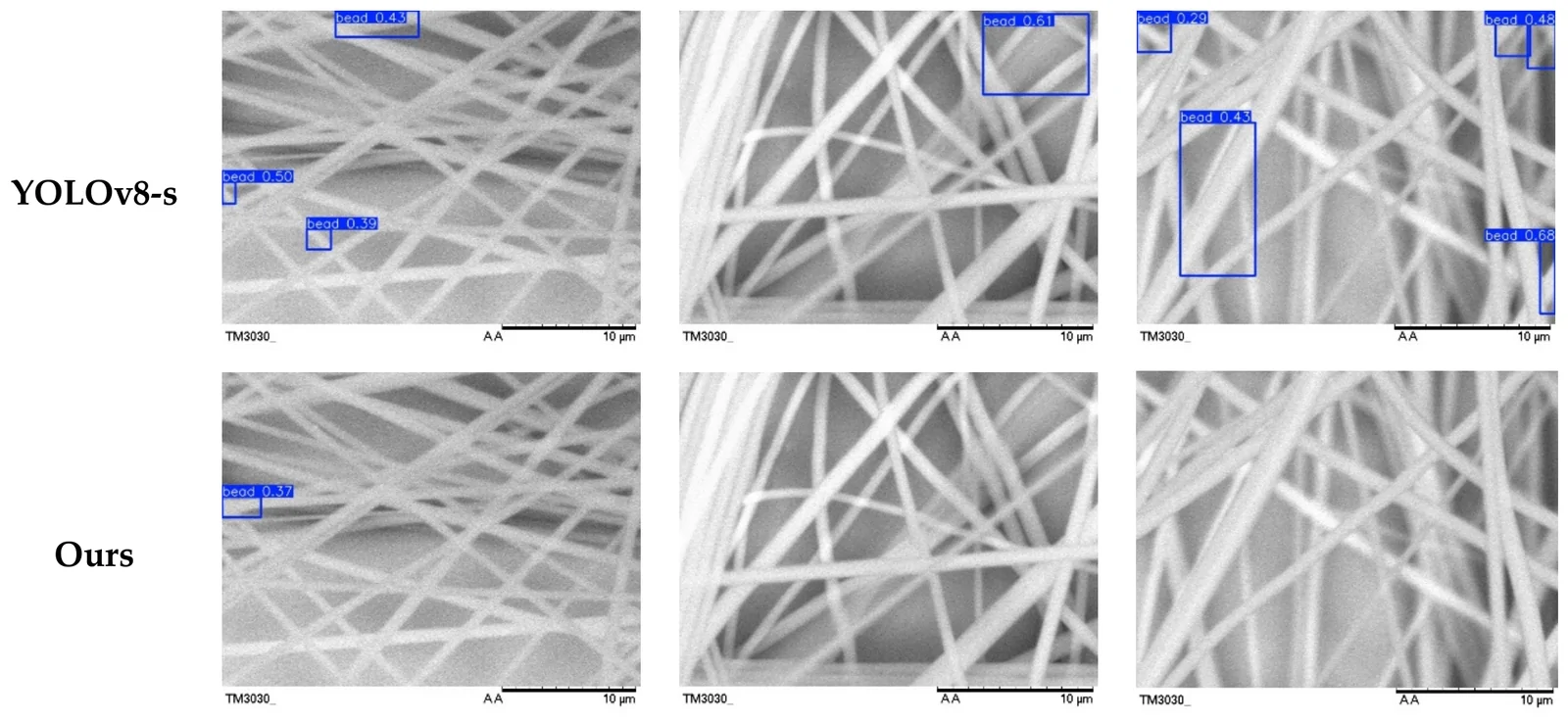
Detection comparison of baseline model and improved model on flawless membrane samples.

The results are presented in Table 5. It can be observed that the improved MA-YOLO model achieves a lower false-positive rate on defect-free membranes, demonstrating superior recognition capability in the presence of complex background interference caused by nanofiber morphologies. As illustrated in Figure 13, three defect-free membrane samples were randomly selected for comparative analysis. The baseline model exhibited varying degrees of false detections across these samples, whereas the proposed model maintained reliable detection performance with significantly improved robustness against false alarms.

### 4.4. Comparative Experiment of Different Models

To comprehensively evaluate the performance of the proposed model, comparative experiments are conducted against 11 mainstream and state-of-the-art object detection models, including single-stage, two-stage, and Transformer-based methods. None of the models used pre-trained weights to ensure a fair comparison. The results are reported in Table 6.

In terms of detection accuracy, the proposed model achieves the best performance with an mAP@0.5 of 91.9%. It not only significantly outperforms lightweight models of the same class, such as YOLOv8-s and YOLOv10-s, but also surpasses medium-sized models, including YOLOv8-m and YOLOv10-m, which have substantially higher parameter and computational costs. These results demonstrate that the proposed strategies tailored for nanofiber defect detection effectively enhance feature discrimination and localization accuracy.

In terms of inference efficiency, the proposed model contains only 9.15 M parameters and requires 30.9 GFLOPs, achieving 119.05 FPS. It maintains a strong balance between accuracy, model compactness, and rapid inference capability, offering a superior trade-off between performance and efficiency compared with all models. Overall, the two-stage Faster R-CNN and the Transformer-based RT-DETR exhibit dual disadvantages in both accuracy and speed in this industrial defect detection scenario, whereas single-stage models of the YOLO series are better aligned with the deployment requirements of end-to-end real-time rapid quality inspection.

**Figure 14:**
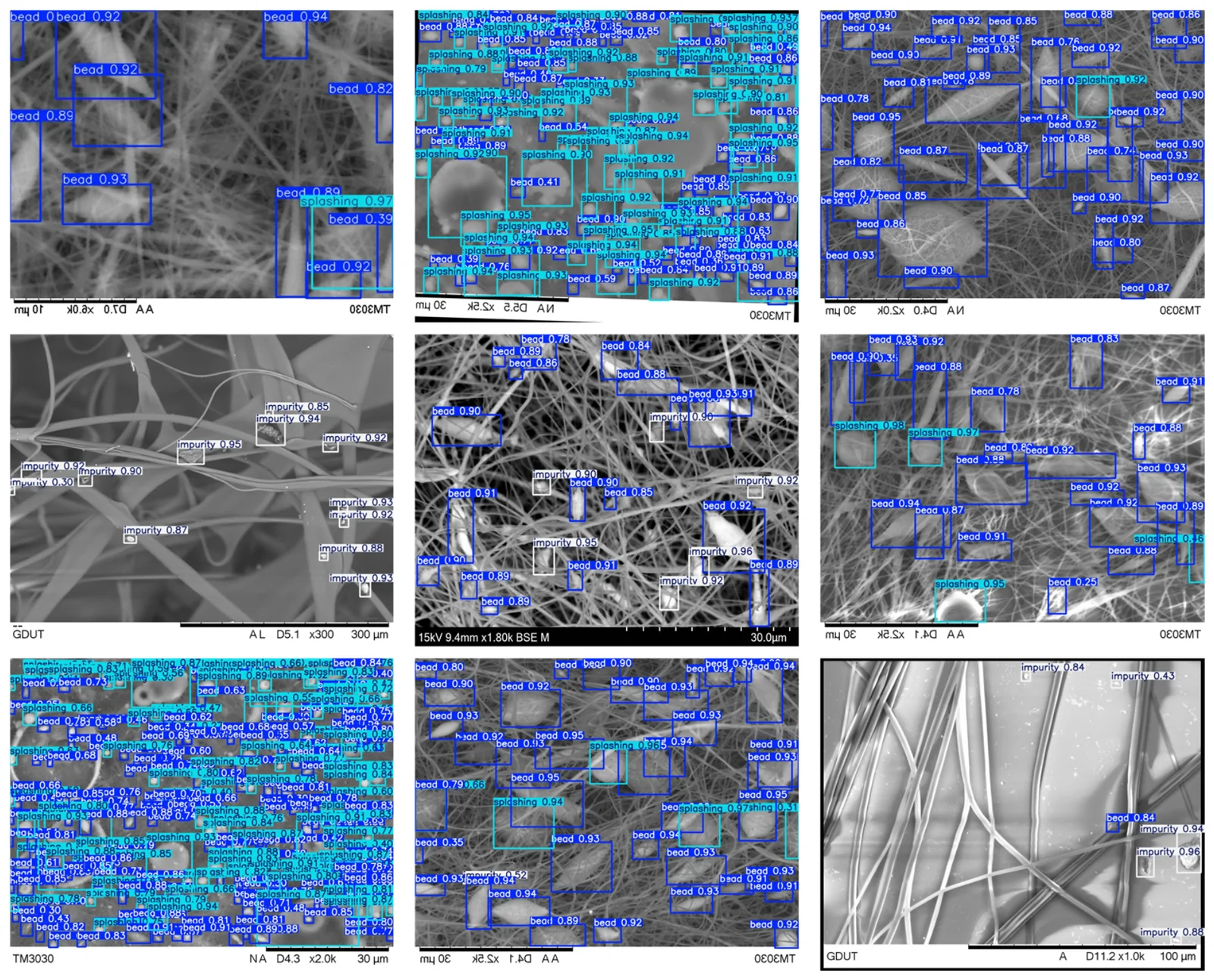
MA-YOLO detection results on nanofiber defect dataset.

**Figure 15:**
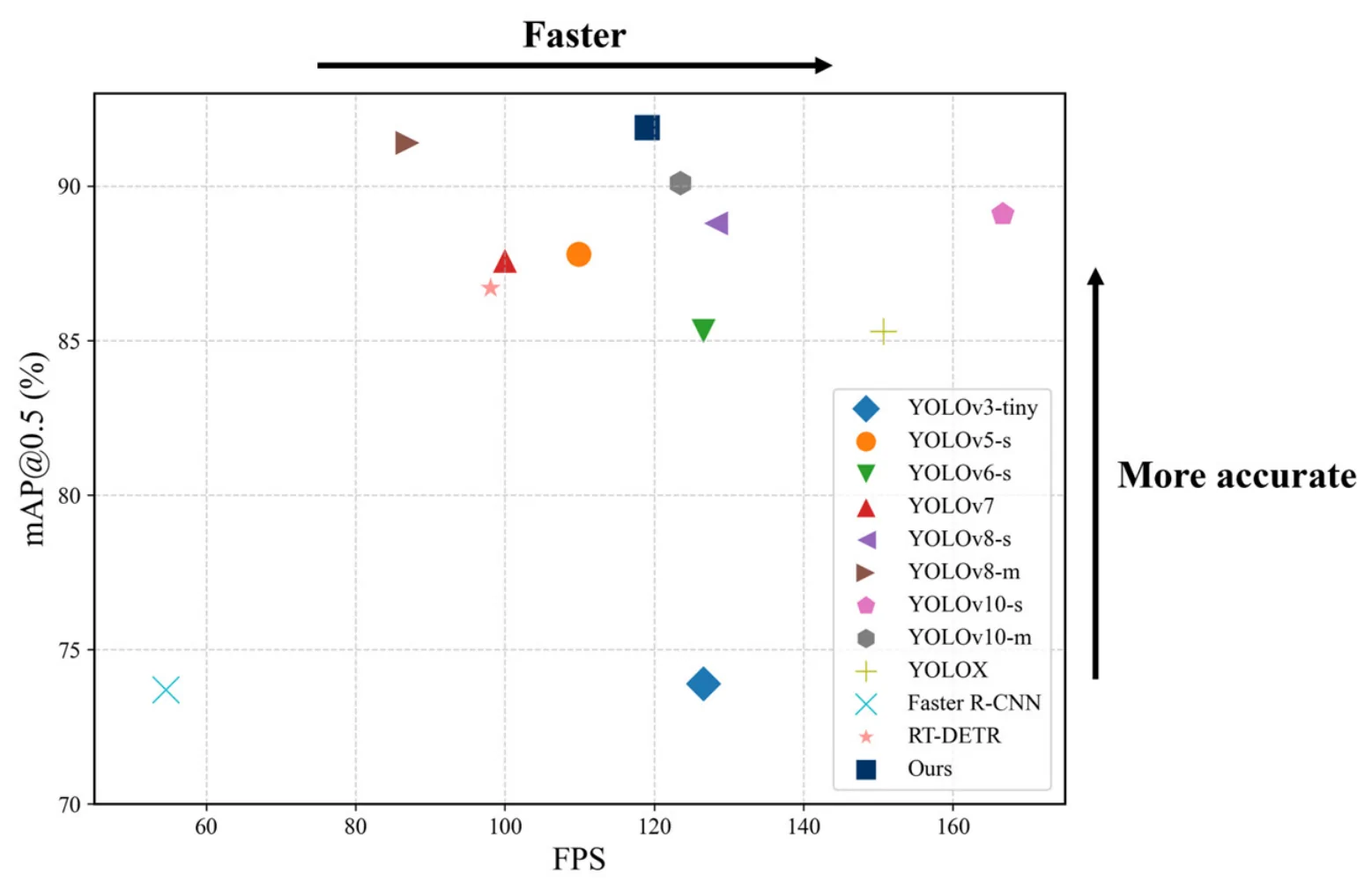
Comparison of accuracy and inference speed across different detection models.

The detection performance of the proposed MA-YOLO model on the nanofiber defect dataset is presented in Figure 14. The varying defect density distributions observed in the figure result from the inclusion of nanofiber membrane images fabricated via different preparation processes in the dataset, reflecting the complex detection scenarios that MA-YOLO is designed to address. Combining the quantitative results above with the visual comparisons in Figure 15, the proposed model demonstrates an optimal trade-off between detection performance and deployment efficiency for nanofiber defect detection.

In practical nanofiber defect detection scenarios, the acquisition time for a single SEM image typically exceeds 120 s. After batch acquisition, images are imported into a detection system integrated with the proposed model for automated inspection. The system achieves an end-to-end full-pipeline frame rate of 73 FPS, corresponding to a single-image processing latency of approximately 13.7 ms. Notably, the per-image detection latency is far shorter than the SEM image acquisition cycle, fully satisfying the demand for rapid inspection. For a typical batch task consisting of 30 SEM images, the model completes full screening within 0.5 s, delivering a substantial improvement in efficiency relative to manual visual inspection. Collectively, these results fully demonstrate the suitability of the proposed model for rapid industrial inspection applications.

## 5. Conclusions

To meet the reliability demands of vibration monitoring in FPD substrate transfer systems and address the critical bottleneck of defect detection of functional nanofiber membranes for flexible vibration sensors, this paper proposes MA-YOLO, a high-precision, rapid defect detection model. By incorporating MLBAN, MA-C2f, MRAF, and IDDH, the proposed model comprehensively enhances defect detection performance across feature fusion, receptive field scaling, morphological adaptation, and task interaction dimensions. Experimental results indicate that the proposed model achieves an mAP@0.5 of 91.9%, representing a 3.1% improvement over the baseline. Under the hardware configuration and parameter settings adopted in this work, it delivers a detection speed of 119.05 FPS and attains an end-to-end full-pipeline frame rate of 73 FPS in practical deployment scenarios, enabling rapid batch quality inspection of nanofiber films. Furthermore, ablation studies validate both the individual contributions and synergistic effects of each module, while cross-model comparisons demonstrate a superior trade-off among detection accuracy, model size, and inference speed.

In the future, we will focus on the application-level link between membrane defects and the performance of flexible sensors, enabling defect detection results to be directly applied to evaluating the reliability of flexible sensors’ monitoring capabilities. At the same time, we will expand the range of defect samples to further enhance the robustness of the detection model.

## Figures and Tables

**Table 1:** Experimental results for different dilation rate combinations in MRAF module.

Dilation Rate	AP Value of Each Category			Precision (%)	Recall (%)	mAP@0.5 (%)	mAP@0.5:0.95 (%)
	Bead (%)	Splashing (%)	Impurity (%)				
[1, 2, 4]	84.1	96.8	85.4	83.4	84.5	88.8	68.2
[2, 3, 5]	84.3	96.8	**89.7**	83.5	86.9	**90.2**	**69.5**
[1, 3, 7]	84	**97.1**	87.7	84	86.6	89.6	69.4
[3, 6, 9]	84.3	96.3	88.6	84	86.9	89.7	68.7
[1, 4, 7]	**84.6**	96	88	83.2	**87.1**	89.5	69.3
[1, 5, 9]	84.4	95.5	88.1	**84.6**	84.6	89.3	68.9

Bold indicates the optimal data in the corresponding column.

**Table 2:** Comparative experimental results of MLBAN and mainstream feature fusion architectures.

Method	AP Value of Each Category			Params (M)	FLOPs (G)	mAP@0.5 (%)	mAP@0.5:0.95 (%)	FPS
	Bead (%)	Splashing (%)	Impurity (%)					
PANet	83.3	96.5	86.5	**11.13**	**28.4**	88.8	68	128.21
BiFPN	**84.0**	95.7	85.2	11.19	28.7	88.3	67.7	**136.99**
MLBAN	83.3	**96.9**	**89.4**	11.91	29.2	**89.9**	**69.1**	133.33

Bold indicates the optimal data in the corresponding column.

**Table 3:** Results of the MA-C2f position ablation experiment.

Method	AP Value of Each Category			Params (M)	FLOPs (G)	mAP@0.5 (%)	mAP@0.5:0.95 (%)	FPS
	Bead (%)	Splashing (%)	Impurity (%)					
(a)	84.5	96.2	87.4	11.29	26.7	89.4	69.5	111.11
(b)	84.5	**97.3**	**87.5**	11.27	27	**89.8**	**70.0**	119.05
(c)	83.3	96.7	85.1	**11.17**	27.8	88.4	68	**126.58**
(d)	84.1	96.4	84.9	11.22	27.6	88.4	67.9	119.05
(e)	**84.9**	94.9	86.5	11.43	**25.2**	88.8	69.1	104.17

Bold indicates the optimal data in the corresponding column.

**Table 4:** Ablation study results for different optimization strategies.

Method	MLBAN	MRAF	MA-C2f	IDDH	Params (M)	FLOPs (G)	mAP@0.5 (%)	mAP@0.5:0.95 (%)	FPS
✓					11.13	28.4	88.8	68.0	128.21
✓	✓				11.91	29.2	89.9	69.1	**133.33**
✓	✓	✓			11.34	28.7	90.4	70.2	131.58
✓	✓	✓	✓		11.48	**27.3**	91.0	71.1	123.46
✓	✓	✓	✓	✓	**9.15**	30.9	**91.9**	**72.0**	119.05

Bold indicates the optimal data in the corresponding column.

**Table 5:** False positive quantitative results of model on flawless membrane.

Model	Defect Boxes	FPI	FPR
YOLOv8-s	84	1.75	0.56
Ours (MA-YOLO)	**51**	**1.06**	**0.40**

Bold indicates the optimal data in the corresponding column.

**Table 6:** Experimental results of different network models.

Model	Backbone	mAP@0.5 (%)	mAP@0.5:0.95 (%)	Params (M)	FLOPs (G)	FPS
YOLOv3-tiny	Darknet53	73.9	39.8	8.67	**12.9**	126.58
YOLOv5-s	CSP-Darknet53	87.8	58.5	**7.02**	15.8	109.89
YOLOv6-s	EfficientRep	85.3	64.4	16.3	44	126.58
YOLOv7	Darknet53	87.6	56.1	15.49	26	100
YOLOv8-s	CSP-Darknet53	88.8	68.0	11.13	28.4	128.21
YOLOv8-m	CSP-Darknet53	91.4	**75.0**	25.84	78.7	86.96
YOLOv10-s	CSP-Darknet53	89.1	67.1	7.22	21.4	**166.67**
YOLOv10-m	CSP-Darknet53	90.1	73.0	15.31	58.9	123.46
YOLOX	Darknet53	85.3	61.0	8.94	13.32	150.72
Faster R-CNN	Resnet50-FPN	73.7	43.1	41.36	167	54.6
RT-DETR	Resnet18	86.7	63.8	19.88	56.9	98.04
Ours	CSP-Darknet53	**91.9**	72.0	9.15	30.9	119.05

Bold indicates the optimal data in the corresponding column.

## Data Availability

Data are available on reasonable request from the corresponding authors.
